# Design of an Isometric End-Point Force Control Task for Electromyography Normalization and Muscle Synergy Extraction From the Upper Limb Without Maximum Voluntary Contraction

**DOI:** 10.3389/fnhum.2022.805452

**Published:** 2022-05-27

**Authors:** Woorim Cho, Victor R. Barradas, Nicolas Schweighofer, Yasuharu Koike

**Affiliations:** ^1^School of Engineering, Tokyo Institute of Technology, Yokohama, Japan; ^2^Institute of Innovative Research, Tokyo Institute of Technology, Yokohama, Japan; ^3^Division of Biokinesiology and Physical Therapy, University of Southern California, Los Angeles, CA, United States

**Keywords:** muscle synergy, EMG normalization, maximum voluntary contraction (MVC), isometric force control, electromyography (EMG)

## Abstract

Muscle synergy analysis *via* surface electromyography (EMG) is useful to study muscle coordination in motor learning, clinical diagnosis, and neurorehabilitation. However, current methods to extract muscle synergies in the upper limb suffer from two major issues. First, the necessary normalization of EMG signals is performed *via* maximum voluntary contraction (MVC), which requires maximal isometric force production in each muscle. However, some individuals with motor impairments have difficulties producing maximal effort in the MVC task. In addition, the MVC is known to be highly unreliable, with widely different forces produced in repeated measures. Second, synergy extraction in the upper limb is typically performed with a multidirection reaching task. However, some participants with motor impairments cannot perform this task because it requires precise motor control. In this study, we proposed a new isometric rotating task that does not require precise motor control or large forces. In this task, participants maintain a cursor controlled by the arm end-point force on a target that rotates at a constant angular velocity at a designated force level. To relax constraints on motor control precision, the target is widened and blurred. To obtain a reference EMG value for normalization without requiring maximal effort, we estimated a linear relationship between joint torques and muscle activations. We assessed the reliability of joint torque normalization and synergy extraction in the rotating task in young neurotypical individuals. Compared with normalization with MVC, joint torque normalization allowed reliable EMG normalization at low force levels. In addition, the extraction of synergies was as reliable and more stable than with the multidirection reaching task. The proposed rotating task can, therefore, be used in future motor learning, clinical diagnosis, and neurorehabilitation studies.

## Introduction

Electromyography (EMG) describes muscle activation by measuring the electrical activity of muscles (Wu et al., 2013). The EMG analysis is widely applied to monitor and evaluate the motor performance of neurotypical individuals (Brueckner et al., 2018; Singh et al., 2018) and the motor function and physiological condition of individuals with neurological deficits (Cheung et al., 2012; Singh et al., 2018; Pan et al., 2021). To measure EMG signals in a non-invasive way, surface EMG is measured by attaching electrodes on the skin. However, surface EMG relies on measurements of the electric activity of muscles through the skin, making it vulnerable to intrinsic and extrinsic factors such as motor unit properties, skin condition, and the placement of the electrode (Hsu et al., 2006; Gaudet et al., 2016). Therefore, raw EMG signals need to be normalized for quantitative comparisons between different muscles and experimental sessions within and between subjects (Burden, 2010).

Maximum voluntary contraction (MVC) is the most commonly used method for EMG normalization (Hsu et al., 2006). During an MVC task, EMG is measured to find the reference values that correspond to a maximal effort contraction (Konrad, 2005). These reference values are then used to normalize the EMG measured during the actual experimental task. However, one of the limitations of the MVC method is that generating maximal effort contractions may be difficult and is affected by muscle fatigue or pain, especially for elderly individuals and individuals with neurological deficits (Hsu et al., 2006; Sousa et al., 2012). Furthermore, because some upper limb muscles are involved in force generation in a direction perpendicular to the reaching plane (Roh et al., 2012), there is a concern that some force would be generated on the perpendicular direction when maximally producing force, even when the task only requires forces contained in the horizontal plan. Last, the reliability of MVC for intersession comparison is affected by the interval between sessions (Kollmitzer et al., 1999), which may be inadequate for monitoring changes in EMG during motor learning or rehabilitation, for instance. Thus, a method for normalizing EMG that does not rely on MVC is needed. EMG normalization is also a necessary processing step for analyses that establish relationships between muscles, such as muscle synergy analysis. Muscle synergies are groups of muscles that the central nervous system (CNS) activates in a coordinated way during the execution of a motor task (Tresch et al., 1999). Therefore, the analysis of muscle synergies is useful for describing and evaluating patterns of muscle activation during a given task (Gentner et al., 2013; Taborri et al., 2018). For example, a muscle synergy can serve as a low dimensional index for motor performance or skill training in neurotypical individuals (Kristiansen et al., 2015), as the muscle synergy may reflect adaptation to changes in neurological or external conditions, appearing as a change in muscle activation patterns (Carson and Riek, 2001; Shemmell et al., 2005; Safavynia et al., 2011; Brueckner et al., 2018; Li et al., 2019). In neurorehabilitation, muscle synergies can serve as a physiological marker of a patient’s motor impairment or to describe the effect of a treatment (Cheung et al., 2012; Hesam-Shariati et al., 2017; Pan et al., 2018, 2021; Singh et al., 2018).

Isometric multidirection reaching tasks are commonly used for extracting synergies of upper extremity muscles (Dewald et al., 1995; Roh et al., 2012; Berger et al., 2013; Gentner et al., 2013; Barradas et al., 2020). In these tasks, subjects are instructed to move a cursor controlled by the subject’s end-point force to targets distributed uniformly around a center point. However, these tasks suffer from two weaknesses for synergy analysis. First, these tasks demand relatively precise force generation in multiple directions, which can be difficult for individuals with motor impairments. As a result, such participants are excluded from the experiment (Dewald et al., 1995), or the missing data are excluded from the synergy analysis (Roh et al., 2013). For instance, in Dewald et al. (1995), ten out of twenty participants were excluded. In both cases, this data removal was prone to bias the results. Second, the validity of the extracted synergies is vulnerable to the number of the reaching directions in these tasks (Augenstein et al., 2020). This is because if only a few discrete directions are used in the task, the EMG data for missing directions must be interpolated, which could affect the shape of muscle synergy tuning curves. A continuous motor task in which the participants generate forces in all directions would be beneficial for the extraction of synergies that better represent the muscle activation patterns of subjects during a reaching task.

For these reasons, we proposed here a new continuous isometric end-point force control task that enables both EMG normalization without relying on an MVC task and muscle synergy extraction without precise force generation in different reaching directions. This new task consists of maintaining a cursor controlled by the arm’s end-point force on a target that rotates at a constant angular velocity around a central position at a constant force magnitude. Therefore, the rotating task has continuous angular information in all directions of the reaching plane in an isometric condition. Additionally, to make the rotating task have looser motor control requirements, the target and the cursor are large and have blurry edges (Burge et al., 2008) to weaken the sense of their exact location, giving subjects fewer constraints to follow the cursor.

The rotating task allows the use of joint torque normalization (Shin et al., 2009) as a replacement for MVC normalization. Joint torque normalization uses the relationship between estimated joint torques from the end-point force and EMG. We showed that low levels of joint torque far from maximal effort levels are sufficient to conduct reliable normalization of the EMG signals. To confirm the reliability of EMG normalization, we compared the results of joint torque normalization using the rotating task and the results of an MVC task. Furthermore, to validate the muscle synergy extraction in the rotating task, we compared the synergy extraction results of the rotating task and a multidirection reaching task.

## Materials and Methods

### Participants

Ten healthy subjects (all males, 28.6 years, SD: 5.4) participated in the experiment. All subjects were right-handed and used their right arm to perform the experimental tasks. Subjects performed the commonly used planar multidirection isometric reaching task, the MVC task, and the proposed rotating task. All experimental procedures were approved by the ethics board of the Tokyo Institute of Technology, and all subjects provided written informed consent before participating in the study.

### Experimental Setup

Subjects sat on a chair facing a computer screen and held a handle attached to a force sensor centered at the body midline. The height of the seat was adjusted so that the sensor and the arm lay on a horizontal plane. The arm’s weight was supported by a two-link elbow support, and the wrist was constrained by a splint. Based on previous studies (Berger et al., 2013), surface EMG signals from 10 shoulder and elbow muscles (i.e., trapezius, posterior deltoid, middle deltoid, anterior deltoid, pectoralis major, triceps brachii long head, biceps long head, triceps brachii lateral head, brachioradialis, and pronator teres) were measured using bipolar electrodes (Bagnoli system; Delsys). The arm end-point force was measured with a 6-axis force sensor (DynPick WEF-6A200; Wacoh-tech). Raw EMG and force data were sampled at 2,000 Hz using an analog-to-digital converter (USB-6363 BNC; National Instruments) and processed and analyzed using Matlab 2020. Filtered end-point force data were projected on a 30-inch LCD screen as a visual feedback cursor, so that subjects could control the cursor position according to the experiment instructions.

### Experiment Protocol

Subjects performed three isometric tasks, namely, the proposed rotating task, the multidirection reaching task, and the MVC task. The tasks were performed on the same day. We grouped the MVC and the multidirection reaching tasks as one task unit, in which the MVC task was always conducted before the multidirection reaching task. The rotating task constituted a separate task unit. We counterbalanced the order of these two task units across subjects to reduce the effects of fatigue and any other task order effects. Furthermore, we asked subjects about their fatigue condition in every task transition to confirm that they were ready to move on to the next task. The next task was only conducted with the subject’s agreement.

#### Rotating Task

**FIGURE 1:**
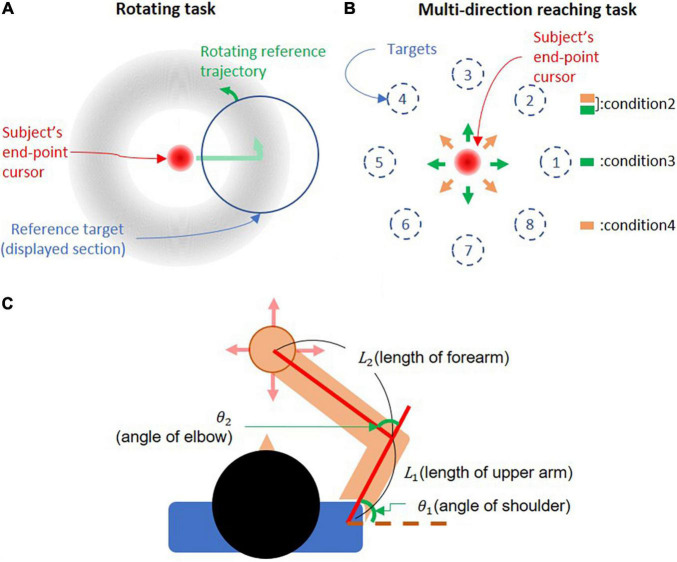
Experimental setup. **(A)** Rotating task. The cursor is controlled by the subject’s end-point force. Subjects move the cursor around a circular trajectory indicated by a doughnut-shaped silhouette. Only a portion of the silhouette enclosed by the reference target is visible during the task. The reference target rotates around the trajectory, setting the pace at which subjects move the cursor. **(B)** Multidirection reaching task. The cursor is controlled by the subject’s end-point force, and targets are presented according to the trial condition (direction and force level). Subjects were instructed to move the cursor to target. **(C)** Subject’s arm posture. The upper arm was set between 40° and 70° with respect to the frontal axis. The forearm was fixed to the handle, so that it formed an angle between 65° and 90° with the upper arm.

In the rotating task, subjects controlled the cursor using their end-point force to keep the cursor near a reference target that followed a reference trajectory. The reference trajectory was circular and was displayed as a section of a doughnut-shaped silhouette, which gradually faded in shades of gray as the distance from the circular trajectory increased. The subjects were informed that the darkest color in the silhouette indicated the reference force level. The reference target was a circular “window” that revealed a section of the reference trajectory as it rotated around the trajectory (Figure 1). The cursor was a large red circle with a color gradient that gradually faded as the distance to the center of the cursor increased. The gradually fading visual feedback of the target and cursor weakened the sense of their exact position, reducing the pressure for precise motor control (Burge et al., 2008). At the beginning of a trial, subjects placed the cursor on the reference target, which prompted the reference target to start rotating. After the reference target completed a whole revolution around the screen, the trial ended, and subjects had to rest for 5 s. Subjects were able to rest for longer if they so desired. Each trial lasted 20 s. There was no condition to pause or stop during the trial even if subjects could not place the cursor on the target. The task is illustrated in Figures 1A,C.

The parameters of each trial were defined by 64 randomized trial conditions designed to prevent muscle co-contraction and motor planning for the next trial. The 64 conditions consisted of combinations of 2 rotating directions (i.e., CW and CCW), 8 uniformly distributed starting positions around the reference trajectory for the reference target, and 4 force levels (i.e., 5, 10, 15, and 20N), which indicated the necessary force to keep the cursor on the circular trajectory. These 64 trial conditions were divided into 8 blocks of 8 trials each, and subjects could rest freely between blocks. For all force levels, the diameter of the cursor was 3.1 cm, and the radius of the reference trajectory was kept at 6.9 cm (in 5, 10, 15, and 20N conditions, a distance of 1 cm in the virtual environment corresponded to 0.72, 1.45, 2.17, and 2.90N, respectively).

#### Maximum Voluntary Contraction Task

The MVC task was used to normalize the EMG signals measured in the multidirection reaching task. We also used the MVC task to normalize the EMG in the rotating task to conduct the muscle synergy analysis for comparison to the multidirection reaching task.

Subjects were instructed to produce the largest possible end-point force in eight uniformly distributed directions. At the beginning of the MVC task, a white ring was displayed at the center of the screen, and subjects moved the cursor into the ring. Later, a solid white circular target appeared in one of the directions, and the subject generated the largest possible force using only the upper limb for 2 s in the indicated direction. The target was set at a distance corresponding to 10N from the center of the virtual environment. The cursor could not move past the target. For applied forces exceeding 10N, the cursor remained at the same distance from the center of the virtual environment. While subjects generated their maximum voluntary force, EMG data were recorded to obtain the maximum EMG values to normalize EMG in the other tasks. This process was repeated twice for each target. Subjects could rest freely between trials.

#### Multidirection Reaching Task

In the multidirection reaching task, subjects controlled the cursor using their end-point force to reach a target that appeared at fixed positions on the screen (corresponding to specific force magnitudes and directions). There were 32 targets in the task, resulting from the combination of 8 uniformly distributed force directions and 4 force magnitudes (5, 10, 15, and 20% of the MVC’s maximum force magnitude). Each target was presented 3 times, resulting in a total of 96 trials. At the beginning of a trial, a white empty circle appeared at the center of the screen, into which subjects moved the cursor. Next, a target was presented according to the trial condition (i.e., direction and magnitude of force), toward which subjects moved the cursor at their preferred speed and timing. If subjects succeeded to reach the target, visual feedback was removed for 2 s. Subjects were instructed to move the cursor to the target and hold the cursor at the target position until visual feedback reappeared. If subjects failed the trial, the trial was repeated. Subjects could rest freely between trials.

For all force levels, the cursor was a solid red circle with a 0.9 cm diameter, and targets were rings with a 4.5 cm diameter to induce precise reaching. The movement of the cursor and applied force from the end point were mapped such that 5% of the MVC’s maximum force magnitude was projected as 3.6 cm on the screen.

### Data Analysis

#### Electromyography Signal Processing

Electromyography data were processed offline. Raw EMG signals were rectified and filtered to obtain a linear envelope of the EMG signal. First, we used a Butterworth second-order high-pass filter with 20 Hz cutoff frequency. Second, the high-pass-filtered data were rectified and filtered with a Butterworth second-order low-pass filter with a 5 Hz cutoff frequency. Finally, the filtered data were normalized using two different methods, namely, MVC normalization and joint torque normalization.

#### End-Point Force Data Processing

##### Rotating Task

In the rotating task, to give visual feedback to subjects, the force was re-sampled at 100Hz and filtered using a moving average filter with a window size of 15 samples. This filter was used to avoid delays, since we found that control in the rotating task is more susceptible to delays than that in the multidirection reaching task. To analyze force trajectories on the plane, the force was filtered using a median filter with a window size of 15 samples. The scale of the force-position mapping on the screen was adjusted so that the size and position of elements on the screen remained constant across all conditions.

##### Maximum Voluntary Contraction Task

In the MVC task, a Butterworth second-order low-pass filter with a 1 Hz cutoff frequency was used to filter the raw force signals. We measured forces in the vertical direction to verify whether maximal force production in the horizontal plane was associated with forces outside of the horizontal plane. We calculated the ratio of the magnitudes of the vertical force and the maximum force in the reaching plane.

##### Multidirection Reaching Task

In the multidirection reaching task, a Butterworth second-order low-pass filter with a 1 Hz cutoff frequency was used for filtering the raw force signals. The filtered force data were scaled to provide the position of the cursor on the screen according to the MVC task’s maximum force magnitude.

### Joint Torque Estimation

For the joint torque normalization, the torques around the elbow and shoulder were estimated from the end-point force using the virtual work principle according to Eqs 1, 2. A two-dimensional model of each subject was used, as seen in Figure 1C. We measured the length of the upper limb segments and the elbow and shoulder joint angles to define the two-dimensional model.


(1) $$\text{J}=\left[\begin{array}{cc} -L_{1}*\sin\left(\theta_{1}\right)-L_{2}*\sin\left(\theta_{1}+\theta_{2}\right) & -L_{2}*\sin\left(\theta_{1}+\theta_{2}\right)\\ L_{1}*\cos\left(\theta_{1}\right)+L_{2}*\cos\left(\theta_{1}+\theta_{2}\right) & L_{2}*\cos\left(\theta_{1}+\theta_{2}\right) \end{array}\right]$$

(2) $$\left[\begin{array}{c} \tau_{s}\\ \tau_{e} \end{array}\right]={\text{J}}^{\text{T}}\times \left[\begin{array}{c} F_{x}\\ F_{y} \end{array}\right]$$

where J is the Jacobian matrix according to a model of the forward kinematics of a planar two-joint arm, *F*_*x*_ and *F*_*y*_ are end-point forces on the reaching plane, and *τ_*s*_* and *τ_*e*_* are the estimated joint torques of the shoulder and elbow.

### Joint Torque Normalization Method

The joint torque normalization method was used to normalize the filtered EMG signals using the relationship between joint torque and muscle activation (Shin et al., 2009). This method finds the relationship between joint torque and filtered EMG by performing a linear regression between these two variables. Next, the estimated filtered EMG value that corresponds to a torque magnitude of 80 Nm in the linear regression model was determined as the reference value for the normalization in each muscle. We followed Shin et al. (2009)’s assumption that 80 Nm is appropriate for estimating the reference value of the joint torque normalization.

To estimate the linear model for the filtered EMG data and joint torque, we only used EMG and torque data corresponding to the anatomical pulling direction of the analyzed muscle, that is, positive torques for joint flexors and negative torques for joint extensors. Then, the filtered EMG data were uniformly divided into 100 bins according to the range of the joint torque data. We defined the representative EMG values of each bin as the 5th percentile of the EMG values in the bin to eliminate surplus EMG activity due to co-contraction. The joint torque value of each bin and representative filtered EMG values were used in the linear regression. The estimated filtered EMG value when the joint torque of the linear function was –80 Nm or 80 Nm, according to the function of each muscle around the joint, was defined as the reference value for joint torque normalization:


(3) $$E\,M\,G_{n\,o\,r\,m}=\frac{E\,M\,G-E\,M\,G_{m\,i\,n}}{E\,M\,G_{r\,e\,f}-E\,M\,G_{m\,i\,n}}$$

where EMG*_*ref*_* is the estimated value of EMG at the 80 Nm torque magnitude, and EMG*_*min*_* is the smallest value of EMG in the whole rotating task trials. Thus, the range of the values of normalized muscle activation goes from 0 to 1.

### Comparison of Normalization Methods

To compare the normalization results of the joint torque normalization method and the results of MVC normalization, we obtained the ratio of the normalization reference values for each muscle using both methods. The ratio was obtained by dividing the reference value obtained from the MVC task by the reference value obtained from the joint normalization method using the data from the rotating task.

### Muscle Synergy Extraction

We used non-negative matrix factorization (non-NMF) to extract muscle synergies in the rotating task and the multidirection reaching task (Lee and Seung, 1999; Berger et al., 2013). For an ideal matrix factorization without residual EMG activity, non-NMF is represented as:


(4) m=Wc

where **m** is a 10-dimensional vector of muscle activations measured during the experiment (normalized EMG by MVC normalization), **W** is a 10×*N* matrix of each muscle activation’s weight vector in the extracted synergy, and **c** is an *N*-dimensional synergy activation vector, where *N* is the number of extracted synergies.

Since ten muscles were measured during the experiment, the possible values of *N* are between 1 and 10. For each value of *N*, we repeated the synergy extraction computation 100 times using a different initial condition for **W** to find the synergy set with the highest reconstruction quality *R*^2^ of muscle activations **m**.

To find the best number of synergies (*N*), *R*^2^ and the slope function of the *R*^2^ curve were used, as in Berger et al. (2013). First, the minimum best synergy number (*N*) was determined when *N* number of synergies in the synergy set could reproduce more than 90% of the EMG data variance. In parallel, *N* was determined as the smallest number for which the slope of a linear regression fit of the *R*^2^ curve between *N* and *N* = 10 had a mean squared error less than 10^–4^ (D’Avella et al., 2006). In case the best synergy numbers *N* calculated using the above two criteria did not match, *N* was chosen as the value for which there was the least overlap between the preferred direction of the extracted synergies. When the best synergy number *N* could not be decided with this process, *N* was fixed to *N* = 4 as the default synergy number of the synergy set.

### Muscle Synergy Extraction Result Comparison

We compared the synergy extraction results of the rotating task and the multidirection reaching task. To compare the synergy extraction result between tasks, we extracted synergies in four conditions. The first condition was the synergy extraction from the rotating task. The second condition was the synergy extraction from the multidirection reaching task. The third and fourth conditions were synergy extractions from reduced data sets of the multidirection reaching task, in which 4 reaching directions were omitted. The third condition comprised the 4 directions aligned with the x- and y-axes of the reaching plane (target numbers: 1, 3, 5, and 7 in Figure 1B), The fourth condition comprised the 4 directions diagonal to the x- and y-axes of the reaching plane (target numbers: 2, 4, 6, and 8 in Figure 1B). These last two conditions helped to analyze the effects of synergy extraction in data sets that are missing information and how the generalizability of synergies may be affected by this.

We evaluated the suitability of the rotating task for synergy extraction by examining the reliability and stability of the synergy extraction results compared with those of the multidirection reaching task. We extracted muscle synergies by fixing the number of synergies from 3 to 5 in each of the 4 conditions defined in the previous section. We then attempted to reconstruct the EMG measured in the multidirection task conditions using synergies extracted from the rotating task and vice versa. We reconstructed the EMG data by estimating synergy activations of a reference synergy set that most adequately reconstructs the muscle activations in the target task:


(5) $${\text{m}}_{r}={\text{W}}_{r\,e\,f}\,{\text{c}}_{e\,s\,t}$$

where **m***_*r*_* are the reconstructed muscle activities, **W***_*ref*_* is the extracted synergy set from the reference task, and **c**_*est*_ are the estimated synergy activations. We obtained **c**_*est*_ by performing a non-negative linear regression between the muscle activations of the target task (**m**) and **W***_*ref*_*. For example, to reconstruct the EMG in the multidirection task with the synergy set of the rotating task, we found **c**_*est*_ by applying a non-negative linear regression between the EMG in the multidirection reaching task and the synergy set of the rotating task. We quantified the goodness of reconstruction in each condition using the *R*^2^ index.

### Statistical Analysis

To find a reliable minimum force level of the rotating task for the joint torque normalization, we compared slopes estimated by joint torque normalization for each force level with slopes estimated using all force levels. We tested the null hypothesis that there would be no difference in estimated slopes between each force level and all force levels by an independent *t*-test (i.e., two-tailed).

To test the reconstruction result of the synergy set in the rotating task and the multidirection reaching task’s conditions, the *R*^2^ indices from the rotating task and each of the multidirection task conditions were compared. We tested the null hypothesis that there would be no difference in *R*^2^ indices between the rotating task and each condition of the multidirection reaching task by using a paired *t*-test (i.e., two-tailed). We also tested the null hypothesis that there would be no difference in the variance of *R*^2^ indices between task conditions by using an *f*-test (i.e., two-tailed). The significance threshold was set to *P* < 0.05. All analyses were performed using MATLAB R2019b.

## Results

### Comparison Between Joint Torque and Maximum Voluntary Contraction Normalization

**FIGURE 2:**
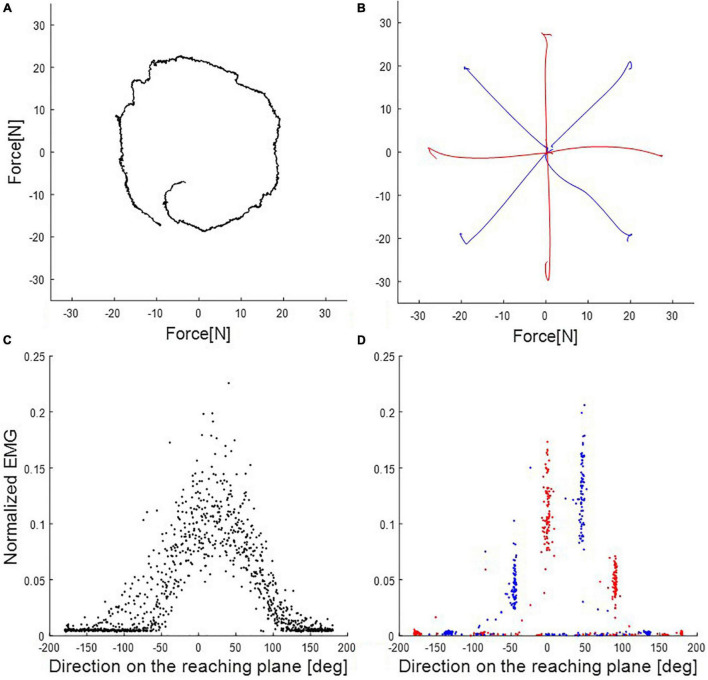
End-point force trajectory and electromyography (EMG) activations in rotating and multidirection reaching tasks of the representative subject (subject 8). **(A)** Rotating task (condition 1) in one trial of 20N trials. **(B)** Multidirection reaching task in trials of 20% of Maximum voluntary contraction’s (MVC’s) maximum magnitude. Condition 2 used all eight targets. Conditions 3 and 4 (red and blue traces, respectively) used subsets of four targets each in different direction. **(C)** EMG activations of the posterior deltoid according to direction on the reaching plane (trials of 20N in the rotating task). Black dots are EMG activation in the rotating task (condition 1). **(D)** EMG activations of the posterior deltoid according to direction on the reaching plane (trials of 20% of the maximum value of MVC in the multidirection reaching task). Red and blue dots are EMG activation in the multidirection reaching task (condition 2). Red and blue dots are subsets, respectively, for conditions 3 and 4.

**FIGURE 3:**
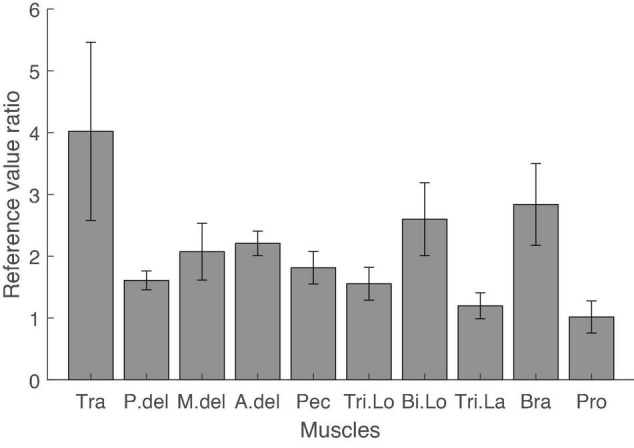
Ratio of reference values of MVC and joint torque normalization. The reference values for EMG normalization of the MVC task and joint torque normalization were compared by dividing the reference value in the MVC task by the reference value in joint torque normalization for each muscle and every subject. The average ratio for every muscle across subjects was larger than 1. The variance of the ratio across subjects was large in trapezius, middle deltoid, biceps long head, and brachioradialis. Error bars indicate the standard error.

**FIGURE 4:**
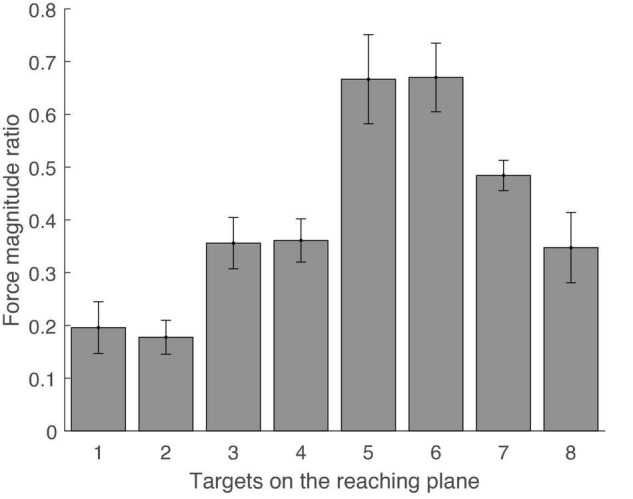
Ratio of magnitudes of perpendicular and in-plane forces for each target during MVC task. Error bars indicate the standard error across subjects.

Figure 2 shows sample trials in the rotating and multidirection reaching task for a representative subject. In the rotating task, subjects produced cursor trajectories with a root mean square error (RMSE) of 12.3% (SD 5.7) of the reference force level with respect to the reference trajectory. All subjects completed all trials of the MVC task and the average resting time between MVC task trials was 4.96 s (SD 3.31, maximum resting time 31 s). We compared the results of joint torque normalization using the rotating task and the results of MVC normalization by calculating the ratio of reference values obtained in both normalization methods (Figure 3). The mean ratio of MVC and joint torque normalization reference values among subjects and muscles was 2.09 (SD 1.95) [Tra: 4.02 (SD 4.56), P.del: 1.61 (SD 0.48), M.del: 2.07 (SD 1.45), A.del: 2.21 (SD 0.63), Pec: 1.81 (SD 0.83), Tri.Lo: 1.55 (SD 0.84), Bi.Lo: 2.60 (SD 1.87), Tri.La: 1.20 (SD 0.66), Bra: 2.84 (SD 2.09), and Pro: 1.02 (SD 0.82)]. Four muscles displayed relatively larger variance in the ratio of reference values than other muscles across subjects (i.e., trapezius, middle deltoid, biceps long head, and brachioradialis). In high effort conditions, such as MVC, these four muscles may be involved in force generation outside of the horizontal plane defined in the isometric tasks. Given that some muscles may generate forces perpendicular to the defined reaching plane, we verified the magnitude of perpendicular forces during the MVC task. We quantified the size of perpendicular forces with respect to planar forces as the ratio of the magnitudes of the perpendicular and reaching plane forces for each target (Figure 4). The mean ratio across all subjects and targets was 0.41 (SE 0.024) [Target1: 0.20 (SE 0.049), Target2: 0.18 (SE 0.032), Target3: 0.36 (SE 0.049), Target4: 0.36 (SE 0.041), Target5: 0.67 (SE 0.084), Target6: 0.67 (SE 0.065), Target7: 0.48 (SE 0.029), and Target8: 0.35 (SE 0.067)]. This indicates that perpendicular forces comprised around 41% of the maximal forces produced on the horizontal plane.

### Minimum Force Level for Joint Torque Normalization

**FIGURE 5:**
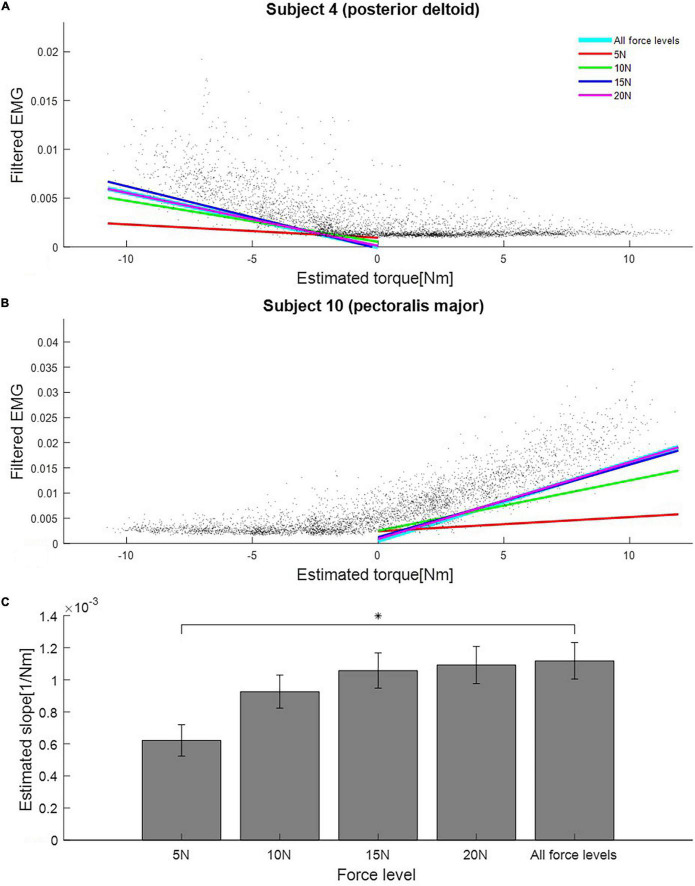
Estimated slopes for joint torque normalization procedure at different force levels in the rotating task. **(A)** Slopes estimated from data of each force level in the rotating task (Subject 4, posterior deltoid). Black dots are samples of estimated torque and EMG. Colored lines represent regression lines between estimated torque and EMG for each force level. **(B)** Slopes estimated from data of each force level in the rotating task (Subject 10, pectoralis major). **(C)** Mean estimated slopes for joint torque normalization for all force levels in the rotating task. Asterisk indicates statistical significance. Error bars indicate the standard error across all muscles and subjects.

We sought to determine the minimum force level that would still produce joint torque normalization results that are comparable to using higher force levels in the rotating task. To do this, we compared the slopes of the regression lines between estimated torque and EMG for every muscle in each level of force in the rotating task. We divided the data set of the rotating task according to each force level and estimated the slope for each force level from the divided data sets. As a result, we found that the slopes estimated for each force level starting from 10N were not statistically different to the slope estimated by combining data from all force levels: [5N: 0.00062[1/Nm] (SE 9.75e–05), *P* = 0.001; 10N: 0.00093[1/Nm] (SE 1.03e–04), *P* = 0.21; 15N: 0.00106[1/Nm] (SE 1.10e–04), *P* = 0.70; 20N: 0.00109[1/Nm] (SE 1.16e–04), *P* = 0.87; all force level: 0.00112 (SE 1.14e–04); independent *t*-test]. The estimated slope for each force level and the corresponding standard error are shown in Figure 5.

### Effect of Number and Direction of Targets on Synergy Extraction

**FIGURE 6:**
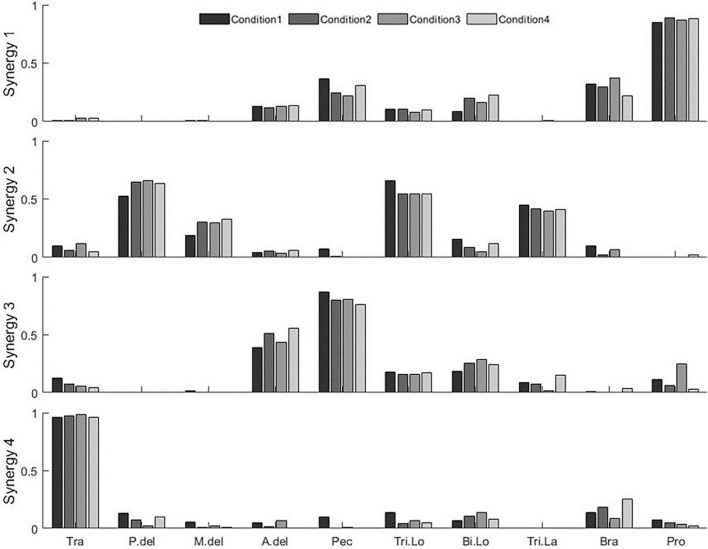
Synergy set extracted in all conditions (condition 1—the rotating task, condition 2—the multidirection reaching task with 8 targets, condition 3—the multidirection reaching task with 4 targets, and condition 4—the multidirection reaching task with 4 targets in diagonal directions) from a representative subject (subject 8).

We compared the results of the synergy extraction procedure using the rotating and the multidirection reaching tasks. We defined two additional sub-tasks based on the multidirection reaching task by varying the number and direction of targets in the tasks. Therefore, we defined four conditions for this analysis (Figures 2A,B): (condition 1: rotating task; condition 2: multidirection reaching task with all 8 targets; condition 3: multidirection reaching task with 4 targets lying on the x- and y-axes of the reaching plane; and condition 4: multidirection reaching task with 4 targets located diagonal to the x- and y-axes on the reaching plane). Figure 6 shows the extracted synergy sets for a representative subject. Table 1 shows the selected number of synergies for each subject and condition. In conditions 1–3, 4–5 synergies were extracted across subjects, whereas in condition 4, 3–5 synergies were extracted. The default synergy number *N* = 4 was used in only 3 out of 40 instances (10 subjects and 4 conditions) of muscle synergy extraction. For conditions 1 and 2, the number of extracted synergies within a subject was the same or fewer in condition 1 except for two subjects (fewer than condition 2 in 5 subjects, same as condition 2 in 3 subjects, and larger than condition 2 in 2 subjects). For conditions 2 and 3, the number of synergies was almost identical (fewer than condition 3 in 1 subject, same as condition 3 in 9 subjects). Finally, for conditions 2 and 4, the number of synergies was larger or the same in condition 2 (same as condition 4 in 3 subjects and larger than condition 4 in 7 subjects). Therefore, this analysis shows that in the multidirection reaching task with a reduced number of targets, synergy extraction was affected according to the directions of the targets.

**TABLE 1:** Number of synergies extracted in each task condition.

Condition	1	2	3	4
	Rotating task	Multidirection reaching task (8 directions)	Multidirection reaching task (4 directions)	Multidirection reaching task (4 directions-diagonal)
Mean (SD)	4.2 (SD 0.42)	4.5 (SD 0.53)	4.6 (SD 0.52)	3.8 (SD 0.63)
Subject 1	4	4	4	4
Subject 2	5	4	5	3
Subject 3	4	5	5	4
Subject 4	4	5	5	4
Subject 5	4	5	5	4
Subject 6	4	4	4	3
Subject 7	5	4	4	3
Subject 8	4	4	4	4
Subject 9	4	5	5	5
Subject 10	4	5	5	4

### Mutual Reconstruction Quality of Muscle Activations in Rotating and Multidirection Reaching Tasks

**FIGURE 7:**
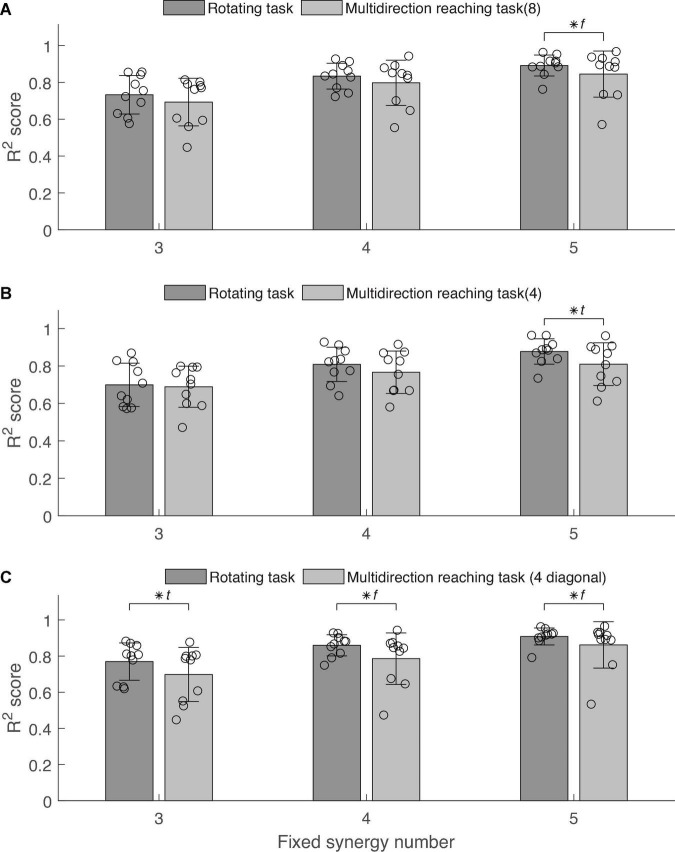
Electromyography reconstruction *R*^2^ score between the rotating task and multidirection reaching conditions. Error bars indicate the standard deviation. Scattered circles indicate *R*^2^ score of each subject in each condition. Asterisk indicates statistical significance in the *t*-test and *f*-test. (A) Comparison between rotating task and multidirection reaching task (8 directions: condition 2). (B) Comparison between rotating task and multidirection reaching task with 4 targets (condition 3). (C) Comparison between rotating task and multidirection reaching task with 4 diagonal targets (condition 4).

We quantified the goodness of reconstruction of the EMG measured in the multidirection task conditions by synergies extracted from the rotating task, and vice versa, by using the *R*^2^ index (Figure 7). We compared the mutual reproducibility of EMG between both tasks. For a fixed number of synergies (i.e., 3–5), synergies extracted from the rotating task produced higher reconstruction qualities *R*^2^ of the EMG in the multidirection reaching task than vice versa. Table 2 contains the results of mutual reconstruction between condition 1 and the other conditions for fixed numbers of synergies and the statistical results. As a general tendency, the *R*^2^ score increased as the number of synergies *N* increased from 3 to 5 in all conditions. The difference in *R*^2^ of the mutual reproducibility between conditions 1 and 2 was not statistically significant. Nonetheless, the standard deviation of *R*^2^ in the reconstruction by the rotating task synergies was smaller than in the reconstruction by the synergies of the multidirection reaching task for *N* = 3–5. The difference in variance between conditions was statistically significant when *N* = 5. In the case of conditions 1 and 3, the difference in *R*^2^ was statistically significant when *N* = 5. However, there was no significant difference in the standard deviation of *R*^2^ between both conditions. Last, in the case of conditions 1 and 4, the reconstruction qualities by the rotating task synergies were higher than the reconstruction qualities by the synergies of the multidirection reaching task for *N* = 3–5. There was statistical significance when *N* = 3. The standard deviation of *R*^2^ in the reconstruction by the rotating task synergies was smaller than in the reconstruction by the synergies of the multidirection reaching task for *N* = 3–5. Furthermore, these differences were statistically significant when *N* = 4 and 5.

**TABLE 2:** The result of electromyography (EMG) activity reconstruction comparison between the rotating task (condition 1) and the multidirection reaching task (conditions 2, 3, and 4) in each fixed synergy number.

Fixed number of synergies		3		4		5	
Rotating task and	mean_*con1*_	0.73 (SD 0.10)	*P*_*t*_ 0.179	0.83 (SD 0.07)	*P*_*t*_ 0.215	0.89 (SD 0.06)	*P*_*t*_ 0.095
Multidirection reaching task (8)	mean_*con2*_	0.69 (SD 0.13)	*P*_*f*_ 0.539	0.80 (SD 0.12)	*P*_*f*_ 0.111	0.85 (SD 0.13)	*P*_*f*_ 0.028

Rotating task and	mean_*con1*_	0.70 (SD 0.12)	*P*_*t*_ 0.762	0.81 (SD 0.09)	*P*_*t*_ 0.131	0.88 (SD 0.07)	*P*_*t*_ 0.012
Multidirection reaching task (4)	mean_*con3*_	0.69 (SD 0.11)	*P*_*f*_ 0.879	0.77 (SD 0.11)	*P*_*f*_ 0.534	0.81 (SD 0.11)	*P*_*f*_ 0.137

Rotating task and	mean_*con1*_	0.77 (SD 0.10)	*P*_*t*_ 0.016	0.86 (SD 0.06)	*P*_*t*_ 0.052	0.91 (SD 0.05)	*P*_*t*_ 0.155
Multidirection reaching task (4 diagonal)	mean_*con4*_	0.70 (SD 0.15)	*P*_*f*_ 0.283	0.79 (SD 0.14)	*P*_*f*_ 0.014	0.86 (SD 0.13)	*P*_*f*_ 0.006
							

*Paired t-test and f-test, bold P-values indicate < 0.05. For statistic comparison of the means of both tasks, non-statistical significance reveals that both tasks are equivalent when we reconstruct EMG activities of other tasks. In case the t-test result has statistical significance, the reconstruction by the rotating task is better because the rotating task has a higher mean than the multidirection reaching task. This implies that the rotating task is as reliable as or, in some cases, more reliable than the multidirection reaching task. For statistic comparison of the variances of both tasks, non-statistical significance reveals that both tasks are equally stable in EMG reconstruction. In case that they are significantly different, the reconstruction by the rotating task is more stable because the variability in the multidirection reaching task is larger than the rotating task in most cases. This suggests that synergies extracted from the rotating task are more stable for reconstructing EMG activities of other tasks.*

In summary, the average *R*^2^ score of the rotating task was equivalent to the *R*^2^ score of other conditions. Moreover, the standard deviation of the *R*^2^ score of the rotating task was smaller than the standard deviation of the *R*^2^ score of other conditions in most cases, reaching statistical significance in 3 cases.

## Discussion

### Muscle Activations Related to Perpendicular Force Generation Produced a Difference Between Maximum Voluntary Contraction Normalization and Joint Torque Normalization

We found that the MVC and joint torque normalization methods produced different results in the normalization reference values. To our knowledge, previous studies examining maximum voluntary isometric force generation in a horizontal plane have not considered the vertical force as a variable of interest. Mainly two-dimensional models and measurements of joint torques and end-point forces are used for tasks on the horizontal plane (Nijhof and Gabriel, 2006; Pinter et al., 2010; Sin et al., 2014; Gorkovenko, 2018; Gorkovenko et al., 2020). Furthermore, in studies that use virtual environments to guide force generation on the two-dimensional plane, there are no reported instructions, feedback, or results related to perpendicular forces during the MVC task (Berger et al., 2013; Gentner et al., 2013; Barradas et al., 2020).

However, our results suggest that the difference between the two normalization methods arose due to sizeable perpendicular forces exerted during the MVC task (Figure 4). On average, the perpendicular force component had a magnitude that was around 41% the size of forces measured on the reaching plane. Consequently, this additional force could have made the MVC’s reference value for some muscles higher than if forces had been applied only on the reaching plane. In particular, because our arm model is defined only for planar forces, increased EMG activity in some muscles due to perpendicular forces would not be associated with corresponding increases in the estimated joint torque. This would appear as a non-linearity in the relationship between estimated joint torque and EMG in the case of the MVC task. Such non-linearities would produce overestimations of the slope of the joint torque-EMG relationship, bringing about a discrepancy with the computed slope in the rotating task. Therefore, we also observed a discrepancy in the ratio of normalization reference values of both tasks across muscles. In other words, since the non-linearities caused by perpendicular forces exerted during the MVC task are different for each muscle, the reference values of MVC normalization and joint torque normalization may be different. However, the difference between the reference values of both normalization methods should be similar between subjects within the same muscle. Therefore, for reliable joint torque normalization between subjects, the variability of the ratio of normalization reference values of both tasks in the same muscle should be small.

### Joint Torque Normalization Allows for Suitable Normalization in Low Force Conditions

Maximum voluntary contraction normalization requires maximal effort muscle contractions that are outside the range of forces produced in daily life. Some of the limitations of this technique are evidenced when applying it to motor-impaired patients, who may be unable to produce the required forces and decreasing reliability due to fatigue and pain (Sousa et al., 2012). Therefore, a reliable EMG normalization method that is easier to perform and improves accessibility is needed. Our results show that using the rotating task at forces as low as 10N can produce joint torque normalization results that are equivalent to results derived from larger forces. Therefore, our results strongly suggest that joint torque normalization in the rotating task enables reliable EMG normalization using low levels of force. This suggests that joint torque normalization is potentially useful as an EMG normalization method for subjects with motor impairments.

### The Rotating Task Is as Reliable as the Multidirection Reaching Task for Synergy Extraction

Extracting synergies that are as general as possible in describing different motor behaviors is important for the analysis of muscle activation patterns. In other words, good synergy extraction is equivalent to extracting synergies that better reproduce various motor behaviors in the same workspace. To confirm the universality of synergies extracted in the rotating task proposed in this study, we tested the ability of these synergies to reconstruct the EMG measured in the multidirection reaching task and sub-tasks and vice versa.

We found that in 7 out of 9 pairings between the rotating task and each condition of the multidirection reaching task, the *R*^2^ mutual reconstruction score was equivalent. In 2 out of 9 pairings, the mean *R*^2^ score was significantly higher for the reconstruction by the rotating task than by the multidirection reaching tasks. This indicates that the mean *R*^2^ score of the reconstruction of EMG in the multidirection reaching tasks by the rotating task across subjects was at least as good as in the reciprocal case. This supports that the generality of synergies in the rotating task was equivalent or better than synergies in the multidirection reaching task. Therefore, the generality of synergies in the rotating task allows the description of the same or more diverse motor behavior information in the reaching plane than multidirection reaching tasks.

Furthermore, we found that in 8 out of 9 pairings between the rotating task and each condition of the multidirection reaching task, the variance of *R*^2^ in the reconstructions by the rotating task was smaller than in the reconstructions by the multidirection reaching tasks. In 3 out of these 8 pairings, the differences in reconstruction variance were significant. This suggests that the stability of EMG reconstruction by synergies in the rotating task was equivalent or higher than by synergies in the multidirection reaching task. Therefore, synergies in the rotating task reconstructed the muscle activations in the reaching plane with higher stability than the multidirection reaching tasks.

We also investigated whether the rotating task elicits muscle activation patterns as complex as the multidirection reaching task by examining the number of synergies extracted in each task. The average number of synergies in the rotating task and the multidirection reaching task were 4.2 (SD 0.42) and 4.5 (SD 0.53), respectively. This indicates that both tasks elicit muscle activation patterns that have a similar degree of complexity. We also investigated whether target number (angular resolution of target distribution on the reaching plane) and target direction affect synergy extraction in the multidirection reaching task. The number of synergies extracted from the multidirection reaching task with 8 directions was usually larger than the number of synergies extracted from the task with diagonal target directions. This suggests that the complexity of the identified EMG activation patterns was higher for the task with a larger number of targets. This may be because the diagonal targets coincide with the directions of action of the shoulder and elbow joints in the examined posture, reducing simultaneous activation of muscles crossing these joints resulting in less complex activation patterns.

Therefore, tasks that rely on discrete reaching movements for synergy extraction are vulnerable to oversimplified muscle synergy descriptions if an inadequate distribution of targets is used. Our results are supported by Augenstein et al. (2020), who indicate that the number of reaching directions and selection of reaching directions in muscle synergy analysis affect the validity of the extracted muscle synergies. Thus, depending on the purpose of the experiment, the target distribution setting on the reaching plane should be carefully considered. This problem is bound to occur when testing subjects with motor impairments, for which some target directions may be excluded in the task or in data analysis due to the inability of the subjects to acquire these targets (Roh et al., 2013), which could bias the synergy analysis. This highlights the benefits of the rotating task, in which the force is generated continuously around the plane, allowing to more granularly identify the area where forces cannot be adequately produced due to subjects’ motor performance. Furthermore, the rotating task allows considerable variability in the magnitude of the produced forces, possibly entailing the production of richer muscle activation patterns.

In conclusion, the rotating task has the potential to replace the multidirection reaching task as a synergy extraction task in the reaching plane of the upper limb. Furthermore, the rotating task has the advantage that EMG data can be normalized with low end-point forces. Additionally, because the area of the plane where patients are not able to adequately execute the task can be more narrowly identified due to the continuous task demands, it may allow to investigate the motor ability of individuals with neurological deficits in higher resolution than the discrete target reaching task. Moreover, one of the ideas of the rotating task was to reduce the pressure for precise motor control to prevent patient’s frustration from failures in motor execution. Therefore, as a future plan, we intend to apply the rotating task to develop a muscle synergy extraction system for patients with motor impairments. In addition, we will compare the influence of the EMG normalization method in the muscle synergy extraction in both neurotypical and neuroatypical individuals. We also intend to extend the rotating task to a three-dimensional task involving force production around a sphere-shaped space, analogous to Roh et al.’s (2012) three-dimensional multidirection reaching task.

## Data Availability Statement

The data presented in this study are available from the corresponding author on reasonable request.

## Ethics Statement

The studies involving human participants were reviewed and approved by Tokyo Institute of Technology Human Subjects Research Ethics Review Committee. The participants provided their written informed consent to participate in this study.

## Author Contributions

WC and VB performed the experiments. WC analyzed data, prepared figures, and drafted the manuscript. All authors conceived and designed the research, interpreted the results of experiments, edited and revised the manuscript, and approved the final version of the manuscript.

## Conflict of Interest

The authors declare that the research was conducted in the absence of any commercial or financial relationships that could be construed as a potential conflict of interest.

## Publisher’s Note

All claims expressed in this article are solely those of the authors and do not necessarily represent those of their affiliated organizations, or those of the publisher, the editors and the reviewers. Any product that may be evaluated in this article, or claim that may be made by its manufacturer, is not guaranteed or endorsed by the publisher.
